# The Spelling of Some Medical Words1Read at the meeting of the American Medical Editors’ Association, in Milwaukee, June 5, 1893.

**Published:** 1893-08

**Authors:** George M. Gould

**Affiliations:** Philadelphia, Pa.


### Selection.

### THE SPELLING OF SOME MEDICAL WORDS.[1]

By GEORGE M. GOULD, A. M., M. D., of Philadelphia, Pa.

(From the *Medical News*, June, 1893.)

1. Of all the languages of the civilized world, there is none that in the most distant manner can rival the English in the ludicrous illogicality and wretched lawlessness of its orthography. In other languages there is a manifest philologic sanity that evidently seeks to hold the written (or printed) word in some sort of relationship with the spoken word. But in our language the reverse seems to be the case ; the more methods in which a single sound can be spelled the better it seemed to please the fathers of the language. As Professor Lounsbury says : "There is nothing more contemptible than our present spelling, unless it be the reasons usually given for clinging to it."

2. The labor which this fact imposes upon the child's mind, and upon all minds that, so far as language-learning goes, persist in the pre-pubertic stage, is a labor that, conceived in its entirety, is literally appalling. The German child learns in one year, and well, what the English child learns in three, and poorly.[2] It is so

1. Read at the meeting of the American Medical Editors' Association, in Milwaukee, June 5, 1893.

2. Professor March says that "it has been computed that we throw away $15,000,000 a year paying teachers for addling the brains of our children with bad spelling, and at least $100,000,000 more paying printers and publishers for sprinkling our books and papers with silent letters."

tremendous a labor that even few educated men reach unconsciousness and ease of orthography, and for the great mass of people it is a constant source of worry or chagrin. To a vast number of people the secret consciousness of their orthographic failing keeps them from the pleasure of writing and composition, or prevents them from profitable employment. To every person that writes, the excess of labor required by our barbaric spelling is a huge waste of time and a heightener of the friction of life. With the correlated barbarism of pronunciation, it is the greatest obstacle to the spread of English as the world's great, sole tongue.

3. The foregoing facts are so incontrovertible that no one who has even cursorily looked into philology and pedagogics has any tendency to deny them. Equally certain is it that all of our great students and masters of philology are entirely agreed as to the tremendous importance of lessening the burdensome labor of education, and the friction of life, by some approach, great or little, toward the phonetic spelling of English words. As succinctly stated in his preface by the learned editor of the great *Century Dictionary*—

> The language is struggling toward a more consistent and phonetic spelling, and it is proper in disputed and doubtful cases to cast the influence of the dictionary in favor of this movement, both by its own usage in the body of the text, and at the head of articles by the order of forms, or the selection of the form under which the word shall be treated.

Never has more capital been invested in similar enterprises, and never has more philologic erudition been gathered to the service, than in the editing and publishing of those splended lexicographic monuments of American scholarship, the *New Webster*, the *Century*, and the *Standard* dictionaries. It is equally true that in each case the most earnest desire of the men in charge of these works has been to go to the furthest admissible limit dared in recommending the shortening and rationalizing of the spelling of English words. They have only stopped when and where they thought further advance would result in a baulking, and a refusal of the people to follow.

Words fail me to express my amazement to hear men object to all change in the customary spelling. To be sure, they are but few, and those who have never given the matter an hour's thought or study, who thus blindly cling to the fetich of custom, stolidly resisting any change whatsoever. The changes that have been made, and that have become the rule—these they willingly accept. They have grown used to spelling *music* and *public* without a final *k*, and are willing to leave off this useless second tail. (The English even now stick to the final *k* in almanac.) But their mental forefathers as stoutly resisted the *curtailing* process, and their similarly-minded children will finally accept the changes that progressive minds are now forcing on their fathers. The stupidest, most disgusting thing in the world, is the brute conservatism that refuses all change, good or not good, from stolid, unreasoning desire for things as they are. Better chorea, ay, better epilepsy than absolute paralysis. Conservatism is the sham coyness of linguistic old-maidism, the crinolin fig-leaf of philologic prudery, a fig-leaf, too, not the result of too much, but of too little knowledge—indeed, of an abysmal ignorance of the history of the language.

And most strange of all is such a dead-blank wall of prejudice on the part of medical men. Their science is a progressive one; their life is harassed and hurried with the crush of duties and opportunities. Every hour's experience teaches them to ignore precedent and to cut by the shortest route to the desired end. No body of men is more hampered, and in no calling is labor so much thwarted as in theirs, by popular inherited prejudices, and the old unsloughed snake-skins of quackery, of myth, and of mummery.

The vast majority of medical words have not grown out of the old languages, either of the ancient living Greek or of the medievally preserved dead Greek. When a word is desired, the modern minter snaps out his Liddell and Scott, gets some words that best suit his purpose, and shakes them together in his etymologic basket until they cohere into some sort of unity, not infrequently a very ludicrous one.

The argument most relied on by the obstructionists is the etymologic one. But even this poor scarecrow cannot be set up in our medical cornfields. I do not think the etymologic argument of much force, even in the general literary language, because already the form in a large portion of our words is altogether misleading, changed, or lost, and because the vast majority of people will and can never know anything of the etymologic rootings of their language. But, far more important still is the fact that with printing came the impossibility of a coinage ever being lost, its history unrecorded, or its tiniest rootlet unpreserved.

But far and away over all is the fact that the needs and the help of the living millions of bodies and minds present and to come outweigh linguistic and philologic considerations. Language was made for man, not man for language.

Moreover, and this note well, despite all the literary coxcombs and philologic old maids of Christendom, reform is inevitable. The people, with unerring instinct, are determined to mold their language into some better conformity to their needs. Slang is riotously rampant, and slang is language in the making. Some reform in spelling is as certain to come as future men and women are certain to come, and wisdom on our part is to accept the inevitable, and to make that inevitable as sensible as we can. As another has said : "The grammarian, the purist, the pernicketty-stickler for trifles is the deadly foe of good English, rich in idioms and racy of the soil."

All this is entirely too long an overture to a very small opera. I wish to beg my brother-editors to accept, and to unite in asking the profession to accept, certain tiny, innocent little changes in a very few of the words they use. Some time ago a valued contributor objected to our editorial suggestion that the *al* at the end of many of our adjectives was a useless length of tail that it were desirable to lop off. He could give no reason except that wonderful reason that it sounded better to say *chemical*, *biological*, *parasitical*, etc., than to say *chemic*, *biologic* and *parasitic*. All argument was useless. I asked him if we should also, in his articles, spell *scientifical*, *basical*, *thermical*, *albuminoidal*, *mesoblastical*, *graphical*, *metrical*, etc., or should we leave off the already-dropped, old simian *al*.

Another valued contributor begged to be allowed to spell *hemorrhage*, *anesthetic*, *orthopedic*, and the like, in the fashion of his ancestors, *i. e.*, with the diphthong. I asked, should we preserve the Greek diphthong in all cases, in *æther*, for example, instead of *ether*, and in hundreds of cases where its retention would make his printed page the object of laughter, even to the etymologic sticklers. "Analogy to the dogs!"—and, of course, logic and argumentation also to the same animals.

After four years of careful investigation and great labor, the American Association for the Advancement of Science has adopted a set of rules for the spelling and pronunciation of chemic terms. Among these rules are those advocating the dropping of the final *e* in all such words as *bromid*, *iodid*, *chlorid*, and the like, and also in all such as *bromin*, *iodin*, *chlorin*, etc. Is there any reason, earthly or unearthly, for not following the suggestion ?

While on the suicidal subject of analogy, reference may be made to the spelling of program. There are people who will use the analogic argument, if it serve their purpose, but forget it when it does not serve them. They will spell *diagram*, *anagram*, etc., without the overlong tail, but they are horrified at *program*. Old Dr. Johnson, in his Contradictionary, spelled some word-endings *our*, others simply *or*. Some of his contradictionary aftercomers stick to his *honour*, *neighbour*, *favour*, and *colour*, though they would not be guilty now of *horrour*, *dolour*, *emperour*, *governour*, etc. They are indignant at meeting *meter* or *center*, but if you ask them to spell *diameter*, *scepter*, *sepulcher*, etc., they are like some other bivalves, they shut up—but "are of the same opinion still."

To conclude: There is not a single argument of value against a moderate and at least a small beginning of some kind of spelling-reform of our intolerable English orthography. As regards the spelling of medical words, any argument has less weight than as regards other words. We owe it to our profession to be progressive in this respect—at least, not to be a dead-weight to the car of progress, and, at the very least, not to pull backward, like an over-obstinate horse, when the wagon (with one *g!*) is pushed on to our heels. Wherefore, brethren, will you not assent to the little advance already gained, and will you not assent to a few little timid steps further? Every argument of logic and uniformity, and every motive of good-will and interest in progress, is on this side.

Why shall we not drop the conjoined letter diphthongs in all words? Let us spell all our words from the Greek *αἷμα*, with the single vowel *e* instead of *æ*. Let us say *hemorrhage*, *hemostatic*, etc., clear through the list. The same with all other *ai's* usually spelled *æ*, as in *orthopedic*, *pediatric*, *anesthetic*. The same with *œ*: Let us accept *edema*, *celiotomy*, *diarrhea*, *fetus*, etc.

Let us adopt, with never a wry mouth, the "American spelling" of *honor*, *center*, *meter* (all the meters and liters!), *program*, and the rest.

Let us get a chart of the rules for spelling chemic terms adopted by the American Association for the Advancement of Science, and hang it in front of our desks, and never spell *iodid*, *sulphid*, *hydrid*, *morphin*, *chlorin*, etc., with more *e's* than we should. It is easier to spell them without the *e's*!

Let us be sensible rather than conservative!

